# Coronary Microvascular Function Assessment using the Coronary Angiography-Derived Index of Microcirculatory Resistance in Patients with ST-segment Elevation Myocardial Infarction Undergoing Primary Percutaneous Coronary Intervention

**DOI:** 10.31083/j.rcm2502069

**Published:** 2024-02-20

**Authors:** Ming Li, Xi Peng, Naixin Zheng, Hu Ai, Ying Zhao, Hui Li, Guojian Yang, Guodong Tang, Fucheng Sun, Huiping Zhang

**Affiliations:** ^1^Department of Cardiology, Beijing Hospital, National Center of Gerontology, Institute of Geriatric Medicine, Chinese Academy of Medical Sciences & Peking Union Medical College, 100730 Beijing, China

**Keywords:** ST-segment elevation myocardial infarction, percutaneous coronary intervention, coronary microvascular function, coronary angiography-derived index of microcirculatory resistance

## Abstract

**Background:**

Studies reporting the status of coronary microvascular function in the infarct-related artery (IRA) after primary percutaneous coronary intervention (PCI) remain limited. This study utilized the coronary angiography-derived index of microcirculatory resistance (caIMR) to assess coronary microvascular function in patients with ST-segment elevation myocardial infarction (STEMI) undergoing primary PCI.

**Methods:**

We used the FlashAngio system to measure the caIMR after primary PCI in 157 patients with STEMI. The primary endpoint was the occurrence of a major adverse cardiovascular event (MACE), defined as a composite endpoint encompassing cardiac mortality, target vessel revascularization, and rehospitalization due to congestive heart failure (CHF), myocardial infarction (MI), or angina.

**Results:**

Approximately 30% of patients diagnosed with STEMI and who experienced successful primary PCI during the study period had a caIMR in the IRA of >40. The caIMR in the IRA was significantly higher than in the reference vessel (32.9 ± 15.8 vs. 27.4 ± 11.1, *p* < 0.001). The caIMR in the reference vessel of the caIMR >40 group was greater than in the caIMR ≤40 group (30.9 ± 11.3 vs. 25.9 ± 10.7, *p* = 0.009). Moreover, the caIMR >40 group had higher incidence rates of MACEs at 3 months (25.5% vs. 8.3%, *p* = 0.009) and 1 year (29.8% vs. 13.9%, *p* = 0.04), than in the caIMR ≤40 group, which were mainly driven by a higher rate of rehospitalization due to CHF, MI, or angina. A caIMR in the IRA of >40 was an independent predictor of a MACE at 3 months (hazard ratio (HR): 3.459, 95% confidence interval (CI): 1.363–8.779, *p* = 0.009) and 1 year (HR: 2.384, 95% CI: 1.100–5.166, *p* = 0.03) in patients with STEMI after primary PCI.

**Conclusions:**

Patients with STEMI after primary PCI often have coronary microvascular dysfunction, which is indicated by an increased caIMR in the IRA. An elevated caIMR of >40 in the IRA was associated with an increased risk of adverse outcomes in STEMI patients undergoing primary PCI.

## 1. Introduction

Primary percutaneous coronary intervention (PCI) remains a standard therapy for ST-segment elevation myocardial infarction (STEMI) patients. However, inadequate myocardial tissue reperfusion can still be observed, despite the success in restoring the epicardial coronary blood flow in the infarct-related artery (IRA). This suboptimal reperfusion could result from coronary microcirculatory injury or dysfunction associated with adverse cardiovascular events [1, 2, 3]. To better describe the multiple pathological mechanisms during myocardial reperfusion, the term coronary microvascular dysfunction (CMVD) has been used in patients with STEMI undergoing primary PCI.

By using a traditional pressure wire and thermodilution technique, measuring the index of microcirculatory resistance (IMR) is currently regarded as the reference standard for assessing the coronary microcirculation status [4, 5]. Pressure‒temperature wire-derived IMR demonstrates high reproducibility and specificity and is not influenced by epicardial stenosis severity or variations in hemodynamic conditions [6]. However, despite being a proven reliable method for assessing microvascular function, pressure–temperature wire-based IMR is not readily available in primary PCI cases owing to its invasive nature.

Alternatively, as an emerging technique for evaluating microvascular function, coronary angiography-derived IMR (caIMR) does not rely on pressure–temperature wires. Previous studies have demonstrated that the pressure‒temperature wire-free method is comparable to pressure‒temperature wire-based IMR, with comparable accuracy, and has been accepted as a widely adopted noninvasive physiological assessment of microvascular function [7, 8, 9]. Studies reporting the status of coronary microvascular function in the IRA after primary PCI are limited. Thus, we aimed to investigate the coronary microvascular function indicated by caIMR and its prognostic implications in patients with STEMI undergoing primary PCI.

## 2. Materials and Methods

### 2.1 Study Population and Primary PCI Procedure

Patients with STEMI admitted to the Beijing Hospital Catheterization Room for primary PCI from January 2020 to December 2022 were prospectively selected. This study was authorized by the institutional ethics committee and carried out in accordance with the principles of the Declaration of Helsinki. STEMI was defined as persistent chest pain lasting for at least half an hour, accompanied by ST-segment elevation of more than 1 mm in 2 or more adjacent leads. Primary PCI was conducted using standard procedures, and the selection of additional interventions (such as manual thrombectomy or glycoprotein IIb/IIIa inhibitors), while stent placement techniques were determined by the treating operator. All patients were treated with a loading dose of aspirin 100–300 mg and clopidogrel 300 mg or ticagrelor 180 mg. Anticoagulation therapy was administered during the primary PCI procedure with weight-adjusted unfractionated heparin or bivalirudin. An automated injector was used during the coronary angiogram procedure. The choice of postprocedural anticoagulation, including low molecular weight heparin and fondaparinux, was at the discretion of the operator and according to the thrombus burden and the risk of stent thrombosis. Multivessel disease (MVD) was defined as stenosis ≥75% of the diameter in at least two major epicardial arteries or their main branches. Left main (LM) disease was defined as left main stenosis ≥50% of the diameter. The ≥75% and ≥50% cutoffs were determined to identify significant stenosis based on visual assessment by at least two experienced operators. The success of the primary PCI was defined as the restoration of final thrombolysis in MI (TIMI) grade 3 or the residual stenosis of IRA ≤20% with stent implantation. The left ventricular ejection fraction (LVEF) was obtained using echocardiography before discharge. The exclusion criteria were as follows: complications of cardiogenic shock, failed primary PCI, poor coronary angiography images, and insufficient angiography view of the IRA and reference vessel.

### 2.2 CaIMR Measurement

**Fig. 1.**
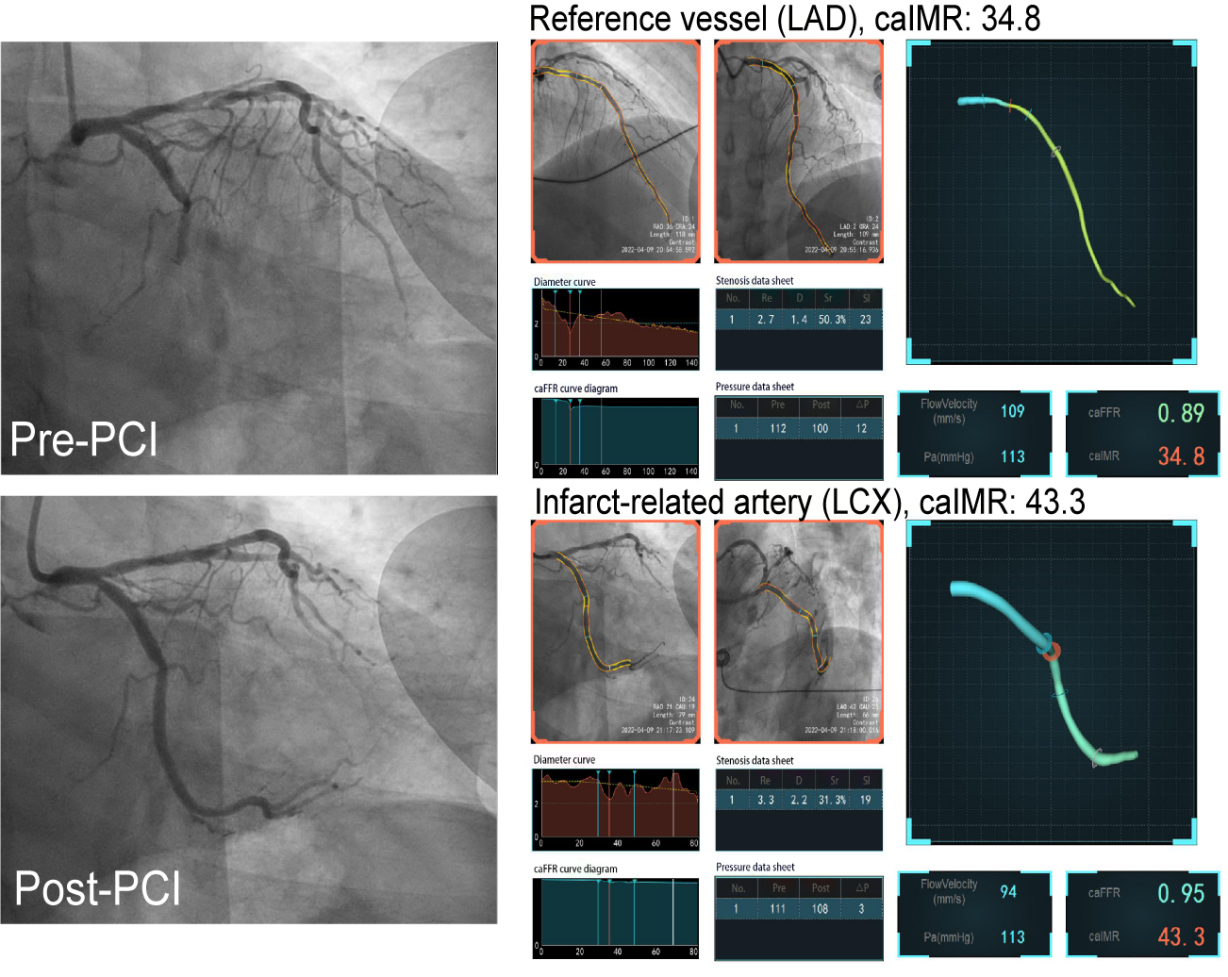
**Representative cases of STEMI with caIMR measurement after primary PCI**. PCI, percutaneous coronary intervention; LAD, left anterior descending artery; LCX, left circumflex; caIMR, coronary angiography-derived index of microcirculatory resistance; STEMI, ST-segment elevation myocardial infarction; caFFR, coronary angiography-derived index of microcirculatory resistance.

CaIMR analysis was performed using the FlashAngio system (Rainmed Ltd., Suzhou, China, Fig. 1). First, a three-dimensional mesh was reconstructed in the target artery using two coronary angiographic projections without overlapping and separated by a minimum 30° angle. Second, aortic pressure was measured using a Flash pressure transducer. Third, several parameters were estimated using computational pressure–fluid dynamics, as previously verified [10]. Hyperemic Pa (${\mathrm{Pa}}_{\text{hyp}}$) signifies the maximal hyperemic mean aortic pressure, calculated by averaging the pressure waves over three cardiac cycles using a mathematical formula described previously [8, 10]. Hyperemic Pd (${\mathrm{Pd}}_{\text{hyp}}$) is the mean distal coronary pressure during maximal hyperemia, calculated using the Navier–Stokes equation. The computational fluid dynamics method was performed to calculate the pressure drop (Δ$P_{\text{hyp}}$) across the meshed coronary arteries, spanning from the inlet to the distal coronary artery.

(1) $$P_{d_{h\,y\,p}}\,\left(\text{ unit: }\,m\,m\,H\,g\right)=P_{a_{h\,y\,p}}-\Delta \,P_{h\,y\,p}$$

L is a nondimensional constant used to represent the distance measured from the inlet to the distal artery. $V_{\text{diastole}}$ (unit: mm/s) represents the average blood flow velocity during diastole, derived via the TIMI frame count method. K is a constant (K = 2.1), K⋅$V_{\text{diastole}}$ represented the maximum hyperemic flow velocity [11, 12]. Finally, the caIMR was calculated as follows:

(2) $$\text{ caIMR }=P_{d_{\text{hyp }}}\,\frac{L}{K\cdot V_{\text{diastole }}}$$

CaIMR was calculated after finalizing the PCI in the IRAs or reference vessels. Reference vessels were designated as nonchronic total occlusion vessels. In patients with severe coronary stenosis, Yong’s formula was used to adjust the caIMR, which accounted for the potential influence of the collateral flow-induced wedge pressure on the caIMR in the presence of substantial epicardial stenosis [13]. Two independent operators were blinded to the clinical information of the patients when performing the measurements. An agreement was reached by consensus when inconsistencies occurred.

### 2.3 Clinical Follow-up

The prespecified primary endpoint was the occurrence of major adverse cardiovascular events (MACEs) at 3 months and 1 year. MACEs were defined as a composite of cardiac death, target vessel revascularization, rehospitalization due to congestive heart failure (CHF), myocardial infarction (MI), or angina. Follow-up was performed through clinic visits, medical record reviews, and phone contact. Survival free of MACEs = (total amount of patients – number of patients with MACEs/total amount of patients) × 100%.

### 2.4 Statistical Analysis

Continuous variables and categorical variables were expressed as mean ± standard deviation or median (interquartile range), as appropriate. Categorical variables are expressed as numbers (percentages). Categorical variables were compared using the chi-squared or Fisher’s exact test. According to distributions, the differences among continuous variables were tested using Student’s *t*-test or the Mann–Whitney rank sum test. Multivariate analysis was performed using a Cox regression model with a stepwise algorithm and expressed as hazard ratios (HRs) with 95% confidence intervals (CIs), to investigate the independent determinants of the primary outcome. Variables related to the outcome of interest were considered as candidate predictors for multivariate analysis based on clinical consideration and demonstrated a *p* value of <0.05 in the univariate analysis. Survival curves for MACE-free outcomes were generated using the Kaplan–Meier method, and group differences were evaluated through the log-rank test. A two-tailed *p* value of <0.05 was considered statistically significant for all tests. All statistical analyses were performed using R version 4.2.2 (R Foundation for Statistical Computing, Vienna, Austria) and Statistical Package for the Social Sciences version 26.0 (IBM Corporation, Armonk, NY, USA).

## 3. Results

### 3.1 Baseline Clinical Characteristics

**Fig. 2.**
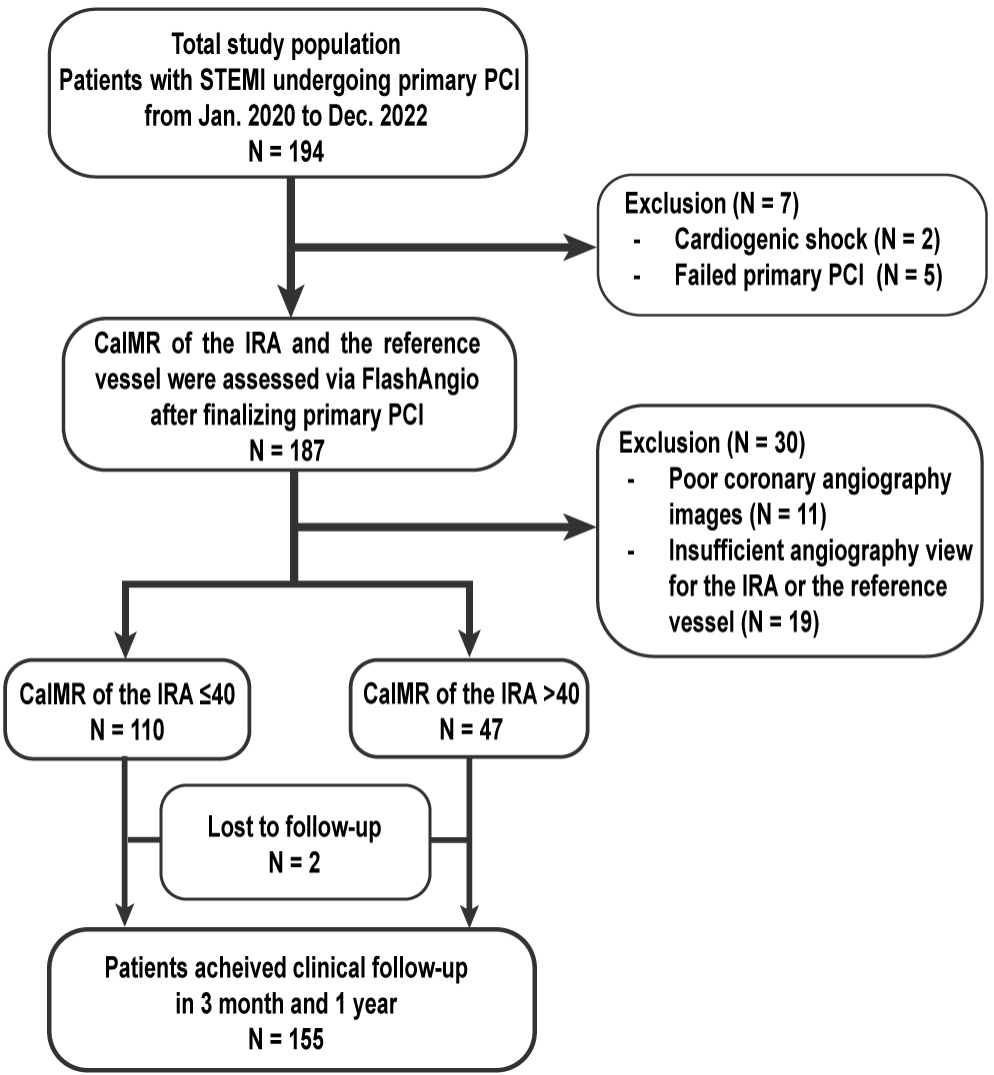
**Study flowchart**. PCI, percutaneous coronary intervention; STEMI, ST-segment elevation myocardial infarction; IRA, infarct-related artery; caIMR, coronary angiography-derived index of microcirculatory resistance.

A total of 194 patients with STEMI who underwent primary PCI were enrolled during the study period. A total of 37 patients were excluded: 2 presented with cardiogenic shock, 5 underwent failed primary PCI, and 30 had poor coronary angiography images and an insufficient angiography view for measurement. Thus, 157 patients were included in the final analysis (Fig. 2). The mean age of the study population was 62.8 ± 14.3 years. Patients were divided into two groups according to the caIMR measurement in the IRA with a cutoff value of 40: the caIMR ≤40 group (70%) and the caIMR >40 group (30%). The detailed clinical characteristics of the patients are summarized in Table 1. Serum peak troponin I (24.4 ± 5.1 vs. 19.4 ± 10.2 ng/mL, *p *= 0.002) and creatine kinase-myocardial band (CK-MB) (233.7 ± 128.7 vs. 157.0 ± 109.9 ng/mL, *p* < 0.001) levels were significantly higher in the caIMR >40 group than in the caIMR ≤40 group. There were no significant differences in any other clinical characteristics between the groups.

**Table 1.** **Baseline clinical characteristics for the study patients**.

		Overall	caIMR ≤40	caIMR >40	*p* value
		n = 157	n = 110	n = 47	
Age (years)		62.8 ± 14.3	62.9 ± 14.3	62.6 ± 14.3	0.93
Male		120 (76.4)	80 (72.7)	40 (85.1)	0.14
BMI (${\mathrm{kg/m}}^{2}$)		25.4 ± 3.9	25.1 ± 3.8	26.1 ± 4.1	0.16
Current smoker		73 (46.5)	51 (46.4)	22 (46.8)	1.00
Hypertension		95 (60.5)	63 (57.3)	32 (68.1)	0.28
Diabetes mellitus		55 (35.0)	40 (36.4)	15 (31.9)	0.72
Dyslipidemia		64 (40.8)	44 (40.0)	20 (42.6)	0.90
WBC (×${\mathrm{10}}^{9}$/L)		10.1 ± 3.0	10.1 ± 3.0	10.1 ± 2.9	0.95
Troponin I peak (ng/mL)		20.9 ± 9.2	19.4 ± 10.2	24.4 ± 5.1	0.002
CK-MB peak (ng/mL)		180.1 ± 120.8	157.0 ± 109.9	233.7 ± 128.7	<0.001
LDL-C (mmol/L)		2.7 ± 1.0	2.8 ± 1.0	2.6 ± 1.1	0.42
LVEF before discharge (%)		47.9 ± 12.1	48.5 ± 12.6	46.4 ± 10.8	0.32
eGFR (mL/min/1.73 $m^{2}$)		85.0 ± 21.5	85.9 ± 22.9	82.9 ± 17.6	0.43
Medication					
	Aspirin	154 (98.1)	108 (98.2)	46 (97.9)	1.00
	Clopidogrel	97 (61.8)	70 (63.6)	27 (57.4)	0.58
	Ticagrelor	59 (37.6)	39 (35.5)	20 (42.6)	0.51
	Statins	148 (94.3)	104 (94.5)	44 (93.6)	1.00
	ACEIs/ARBs	79 (50.3)	57 (51.8)	22 (46.8)	0.69
	Beta blocker	114 (72.6)	78 (70.9)	36 (76.6)	0.59

Values are mean ± standard deviation, n (%), or median (interquartile range). caIMR, coronary angiography-derived index of microcirculatory resistance; BMI, body mass index; WBC, white blood cells; CK-MB, creatine kinase-myocardial band; LDL-C, low-density lipoprotein cholesterol; LVEF, left ventricular ejection fraction; eGFR, estimated glomerular filtration rate; ACEI, angiotensin-converting enzyme inhibitor; ARB, angiotensin II receptor antagonist.

### 3.2 Angiographic and Procedural Characteristics

**Fig. 3.**
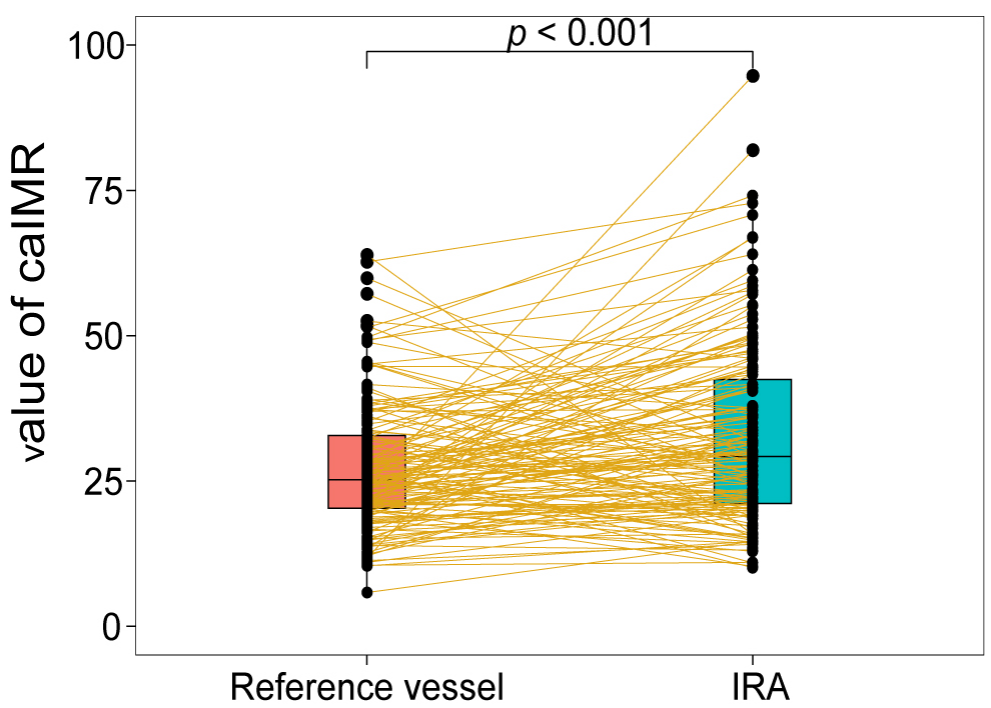
**Paired boxplot between the caIMR in the IRA and reference vessel**. caIMR, coronary angiography-derived index of microcirculatory resistance; IRA, infarct-related artery.

**Fig. 4.**
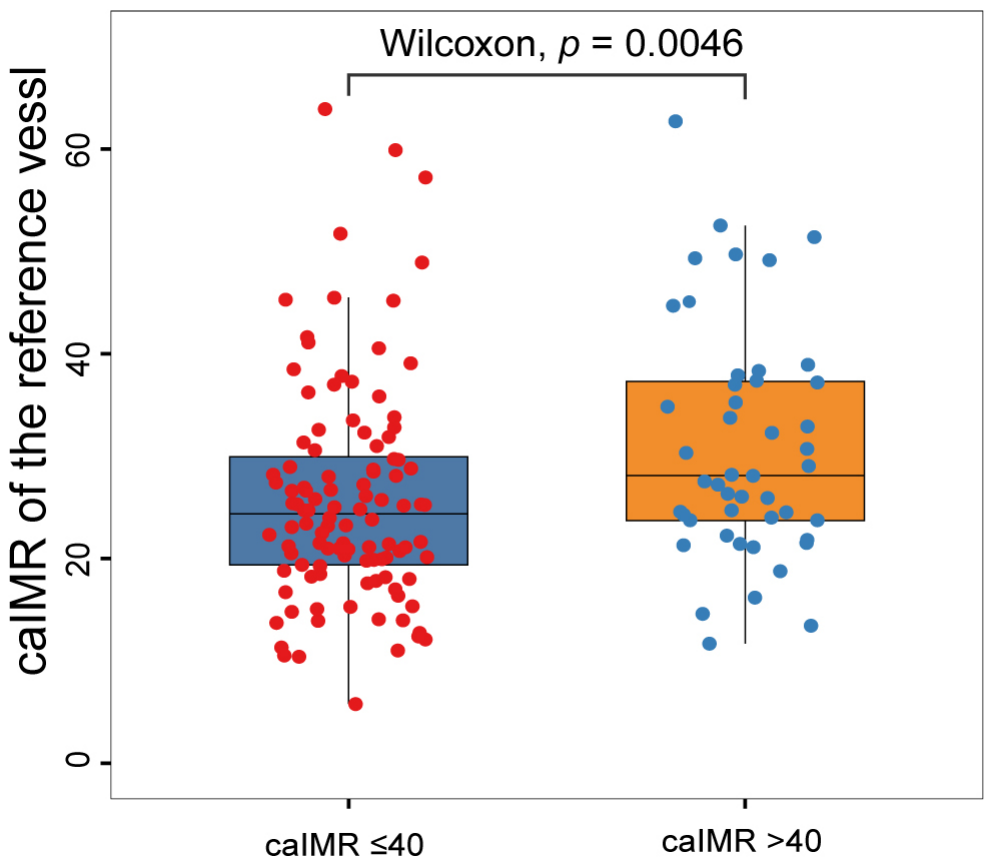
**Distribution of caIMR in the reference vessel according to the caIMR in the IRA**. caIMR, coronary angiography-derived index of microcirculatory resistance; IRA, infarct-related artery.

The angiographic and procedural characteristics are presented in Table 2. The door-to-balloon time was comparable between the groups. The left anterior descending artery (54.1%) was the primary culprit vessel, followed by the right coronary artery (30.6%). The proportion of patients with MVD or LM disease was similar in both groups and presented a similar distribution in the IRA. More patients received thrombus aspiration (31.8% vs. 57.4%, *p *= 0.005) and tirofiban administration (8.2% vs. 25.5%, *p *= 0.008) in the caIMR >40 group than in the caIMR ≤40 group. The proportions of drug-coated balloon use and drug-eluting stent implantation were similar between the two groups. All patients in the caIMR ≤40 group achieved a TIMI grade 3 post-primary PCI, whereas the proportion of patients with the restoration of final TIMI grade 3 was 91.5% in the caIMR >40 group (*p *= 0.002). The caIMR in the IRA was significantly higher than in the reference vessel (32.9 ± 15.8 vs. 27.4 ± 11.1, *p* < 0.001) (Fig. 3). However, the caIMR in the reference vessel for the caIMR >40 group was greater than for the caIMR ≤40 group (30.9 ± 11.3 vs. 25.9 ± 10.7, *p *= 0.009) (Fig. 4).

**Table 2.** **Angiographic, procedural characteristics, and clinical outcomes**.

			Overall	caIMR ≤40	caIMR >40	*p* value
			n = 157	n = 110	n = 47	
Angiographic and procedural characteristics						
	SBP (mmHg)		122 ± 23	120 ± 23	126 ± 21	0.13
	DBP (mmHg)		72 ± 14	70 ± 14	77 ± 14	0.01
	Door-to-balloon time (min)		125 (89, 175)	122 (88, 177)	130 (90, 167)	0.83
	Culprit vessel					0.11
		LAD	85 (54.1)	58 (52.7)	27 (57.4)	
		LCX	21 (13.4)	11 (10.0)	10 (21.3)	
		RCA	48 (30.6)	39 (35.5)	9 (19.1)	
	Multivessel disease		84 (53.5)	58 (52.7)	26 (55.3)	0.77
	LM disease		10(6.4)	6 (5.5)	4 (8.5)	0.49
	Thrombus aspiration		62 (39.5)	35 (31.8)	27 (57.4)	0.005
	Drug-coated balloon use		36 (22.9)	25 (22.7)	11 (23.4)	1.00
	Drug-eluting stent implantation		119 (75.8)	83 (75.5)	36 (76.6)	1.00
	Medication during the procedure					
		Tirofiban	21 (13.4)	9 (8.2)	12 (25.5)	0.008
		Nicorandil	10 (6.4)	5 (4.5)	5 (10.6)	0.28
	Final TIMI flow grade 3		153 (97.5)	110 (100)	43 (91.5)	0.002
	caIMR in the IRA		32.9 ± 15.8	24.3 ± 7.3	52.6 ± 12.0	0.002
	caIMR in the reference vessel		27.4 ± 11.1	25.9 ± 10.7	30.9 ± 11.3	0.009
Clinical outcomes			n = 155	n = 108	n = 47	
	MACEs at 3 months		21 (13.5)	9 (8.3)	12 (25.5)	0.009
		Cardiac death	8 (5.2)	6 (5.6)	2 (4.3)	1.00
		TVR	3 (1.9)	1 (0.9)	2 (4.3)	0.22
		Rehospitalization	10 (6.5)	2 (1.9)	8 (17.0)	0.001
	MACEs at 1 year		29 (18.7)	15 (13.9)	14 (29.8)	0.04
		Cardiac death	8 (5.2)	6 (5.6)	2 (4.3)	1.00
		TVR	5 (3.2)	2 (1.9)	3 (6.4)	0.16
		Rehospitalization	16 (10.3)	7 (6.5)	9 (19.1)	0.02

Values are mean ± standard deviation, n (%) or median (interquartile range). caIMR, coronary angiography-derived index of microcirculatory resistance; SBP, systolic blood pressure; DBP, diastolic blood pressure; LAD, left anterior descending artery; LCX, left circumflex; RCA, right coronary artery; LM, left main; TIMI, thrombolysis in myocardial infarction; IRA, infarct-related artery; MACEs, major adverse cardiac events; TVR, target vessel revascularization; Rehospitalization, rehospitalization for coronary heart failure, myocardial infarct, and angina.

### 3.3 Clinical Outcomes

**Fig. 5.**
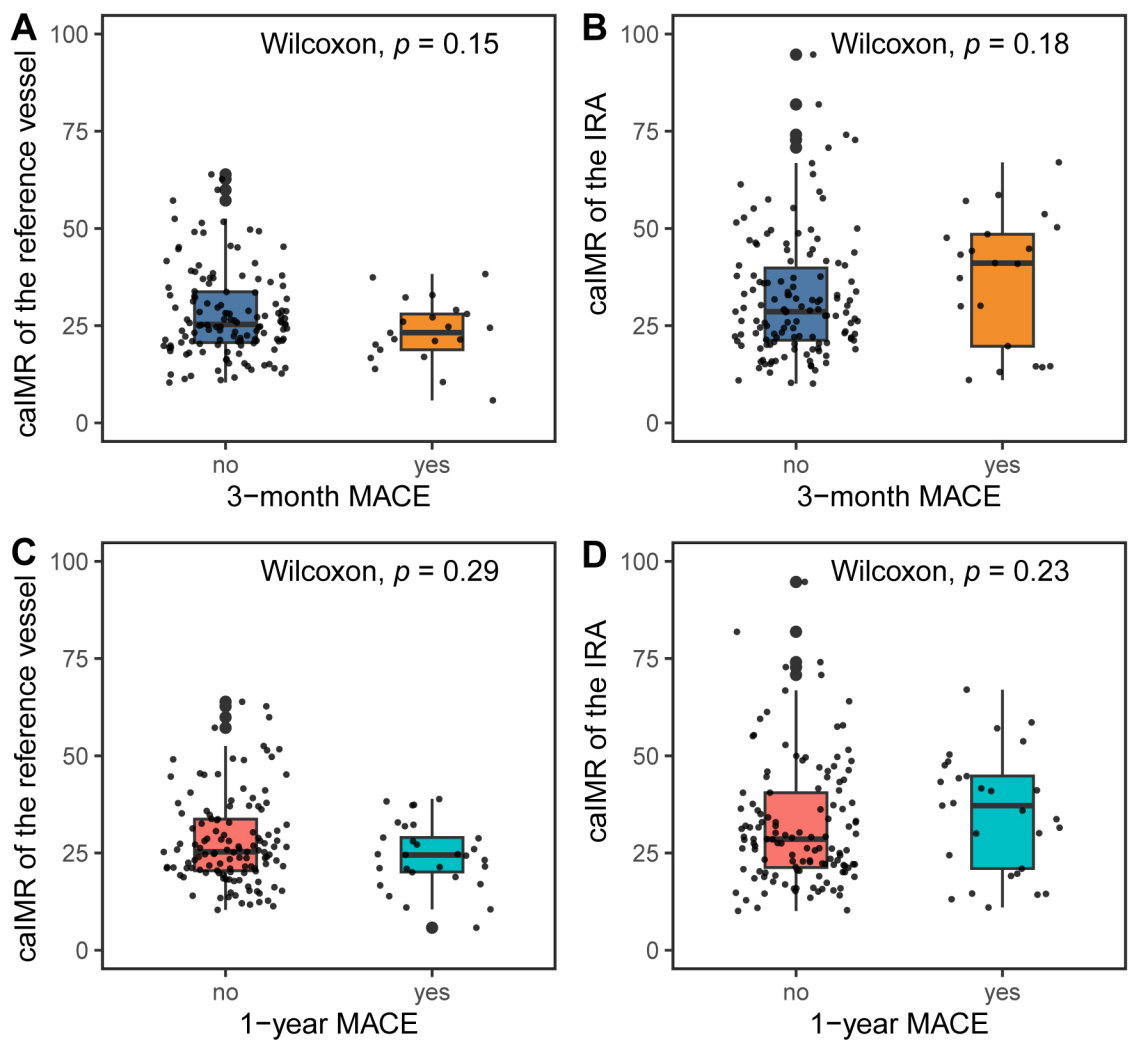
**CaIMR in the IRA and reference vessel according to MACEs**. (A) CaIMR in the reference vessel according to MACEs at 3 months. (B) CaIMR in the IRA according to MACEs at 3 months. (C) CaIMR in the reference vessel according to MACEs at 1 year. (D) CaIMR in the IRA according to MACEs at 1 year. MACEs, major adverse cardiac events; caIMR, coronary angiography-derived index of microcirculatory resistance; IRA, infarct-related artery.

**Fig. 6.**
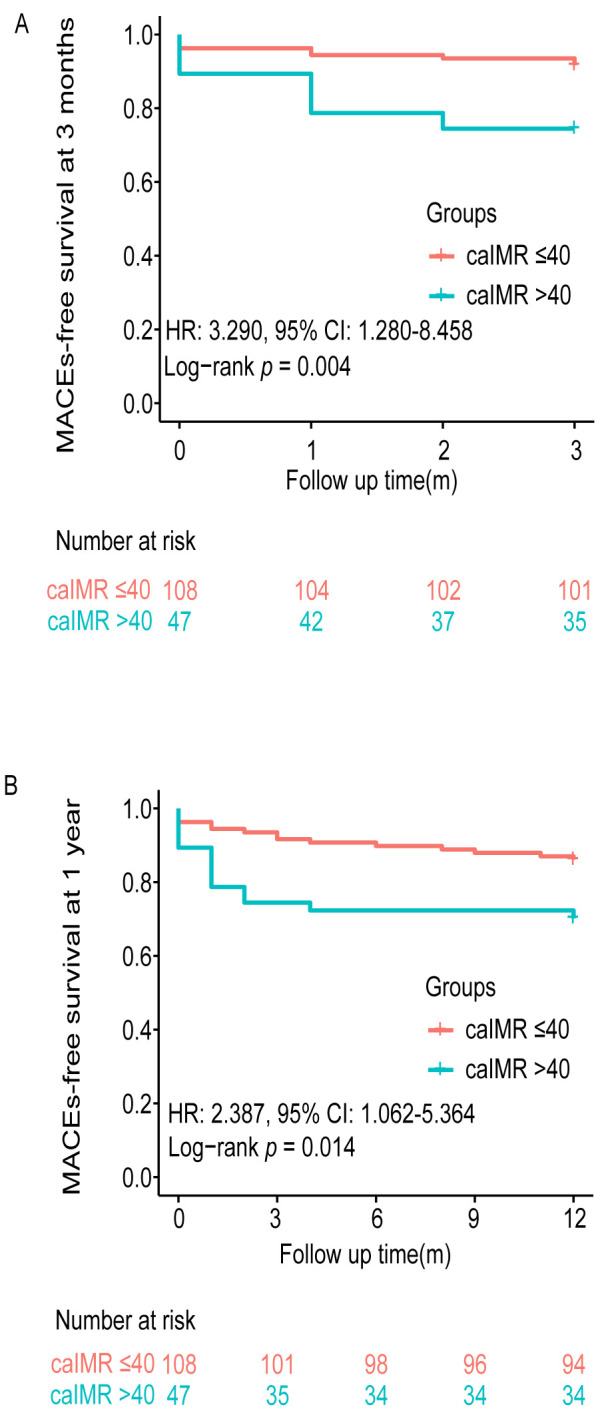
**Kaplan–Meier curves for MACE-free survival according to the caIMR in the IRA**. (A) Curve for MACE-free survival at 3 months. (B) Curve for MACE-free survival at 1 year. caIMR, coronary angiography-derived index of microcirculatory resistance; IRA, infarct-related artery; MACE, major adverse cardiac event; HR, hazard ratio; CI, confidence interval.

The caIMR in the IRA or the reference vessel was comparable between patients with and without MACEs at 3 months and 1 year (Fig. 5). Table 2 displays the clinical outcomes observed at the 3-month and 1-year follow-ups. The caIMR >40 group had higher incidence rates of MACEs at the 3-month (25.5% vs. 8.3%, *p *= 0.009) and 1-year (29.8% vs. 13.9%, *p *= 0.04) follow-ups than the caIMR ≤40 group, which were mainly driven by a higher rate of rehospitalization due to CHF, MI, or angina. Fig. 6 illustrates the MACE-free survival curves at 3 months and 1 year according to whether the caIMR in the IRA was >40.

### 3.4 Predictors of MACEs at 3 Months and 1 Year via Cox Regression Analysis

Candidate predictors found in the univariate analysis included caIMR in the IRA, caIMR in the reference vessel, caIMR in the IRA of >40, age, female sex, hypertension, diabetes mellitus, dyslipidemia, current smoking status, estimated glomerular filtration rate (eGFR), LVEF before discharge <50%, MVD, door-to-balloon time, thrombus aspiration, and final TIMI flow grade 3. The final variables entered into the Cox regression model were caIMR in IRA of >40, age, female sex, hypertension, eGFR, and LVEF before discharge <50%. Tables 3,4 show the multivariate predictors of MACEs at 3 months and 1 year in patients with STEMI who underwent primary PCI. Multivariate Cox regression analysis revealed that a caIMR in the IRA of >40 was an independent predictor of MACEs in patients with STEMI who underwent primary PCI at 3 months (hazard ratio (HR): 3.459, 95% CI: 1.363–8.779, *p* = 0.009) and 1 year (HR: 2.384, 95% CI: 1.100–5.166, *p* = 0.03). Hypertension was also identified as an independent predictor of MACEs at 1 year (HR: 4.026; 95% CI: 1.144–14.162, *p *= 0.03).

**Table 3.** ** Univariate and multivariate analysis of MACEs at 3 months**.

Variables	Univariate Analysis			Multivariate Analysis		
	HR	95% CI	*p* value	HR	95% CI	*p* value
caIMR in the IRA	1.017	0.994–1.041	0.16			
caIMR in the reference vessel	0.959	0.914–1.006	0.09			
caIMR in the IRA >40	3.398	1.431–8.068	0.006	3.459	1.363–8.779	0.009
Age	1.042	1.008–1.077	0.02	1.006	0.967–1.045	0.77
Female sex	2.627	1.107–6.237	0.03	2.079	0.741–5.826	0.16
Hypertension	4.181	1.232–14.197	0.02	2.161	0.570–8.191	0.26
Diabetes mellitus	0.910	0.367–2.255	0.84			
Dyslipidemia	0.573	0.222–1.478	0.25			
Current smoker	2.285	0.886–5.889	0.09			
LVEF before discharge <50%	3.566	1.306–9.736	0.01	2.075	0.696–6.186	0.19
eGFR	0.974	0.957–0.992	0.006	0.981	0.959–1.003	0.09
Multivessel disease	1.807	0.729–4.478	0.20			
Door-to-balloon time	1.002	0.998–1.007	0.25			
Thrombus aspiration	0.733	0.296–1.815	0.50			
Final TIMI flow grade 3	0.510	0.069–3.804	0.51			

MACEs, major adverse cardiac events; HR, hazard ratio; CI, confidence interval; caIMR, coronary angiography-derived index of microcirculatory resistance; IRA, infarct-related artery; LVEF, left ventricular ejection fraction; eGFR, estimated glomerular filtration rate; TIMI, thrombolysis in myocardial infarction.

**Table 4.** **Univariate and multivariate analysis of MACEs at 1 year**.

Variables	Univariate Analysis			Multivariate Analysis		
	HR	95% CI	*p* value	HR	95% CI	*p* value
caIMR in the IRA	1.011	0.990–1.033	0.29			
caIMR in the reference vessel	0.970	0.934–1.008	0.12			
caIMR in the IRA >40	2.446	1.180–5.070	0.02	2.384	1.100–5.166	0.03
Age	1.034	1.006–1.063	0.02	0.999	0.967–1.032	0.96
Female sex	2.584	1.234–5.415	0.01	2.089	0.864–5.047	0.10
Hypertension	6.286	1.902–20.773	0.003	4.026	1.144–14.162	0.03
Diabetes mellitus	1.123	0.530–2.378	0.76			
Dyslipidemia	0.632	0.288–1.389	0.25			
Current smoker	1.780	0.828–3.830	0.14			
LVEF before discharge <50%	2.544	1.158–5.589	0.02	1.738	0.742–4.073	0.20
eGFR	0.978	0.963–0.993	0.006	0.987	0.969–1.005	0.15
Multivessel disease	0.891	0.421–1.887	0.76			
Door-to-balloon time	1.001	0.997–1.005	0.55			
Thrombus aspiration	1.288	0.615–2.697	0.50			
Final TIMI flow grade 3	0.335	0.080–1.410	0.14			

MACEs, major adverse cardiac events; HR, hazard ratio; CI, confidence interval; caIMR, coronary angiography-derived index of microcirculatory resistance; IRA, infarct-related artery; LVEF, left ventricular ejection fraction; eGFR, estimated glomerular filtration rate; TIMI, thrombolysis in myocardial infarction.

## 4. Discussion

This study aimed to evaluate the coronary microvascular function indicated by caIMR in patients with STEMI undergoing primary PCI. The main findings of our study are as follows: (1) CaIMR in the IRA of >40 accounted for approximately 30% of STEMI patients who met the inclusion criteria and underwent successful primary PCI during the study period. Patients with a caIMR in the IRA of >40 experienced obvious myocardial damage compared with those with a caIMR in the IRA of ≤40. (2) There was a significant difference in the coronary microvascular function between the culprit vessel and the reference vessel after primary PCI, as indicated by a higher caIMR in the IRA than in the non-IRA; the caIMR in the reference vessels with a caIMR >40 group was greater than in the caIMR ≤40 group. (3) A caIMR in the IRA of >40 was identified as an independent predictor of short-term and long-term MACEs in patients with STEMI undergoing primary PCI.

Achieving a TIMI grade 3 flow in the IRA is the principal objective of primary PCI, and reperfusion at the myocardial tissue level, manifested by the IMR value, is increasingly important [14, 15]. Owing to the extra procedure time, discomfort in patients resulting from adenosine infusion, the risks associated with manipulating the pressure wire, and additional cost, the use of pressure‒temperature wire-based IMR has limited applications in STEMI. Some studies have indicated that caIMR is a promising and reproducible alternative to wire-based IMR for determining quantitative coronary microvascular function [8, 9, 16]. Since multiple factors are associated with CMVD in primary PCI, the elevation of caIMR in the IRA could be revealed. In this study, the proportion of patients with a caIMR in the IRA of >40 was similar to in early studies, which indicated that approximately one-third of patients who underwent successful primary PCI in the IRA could still be subject to insufficient myocardial perfusion due to CMVD and identified by a caIMR of >40 [4, 5]. Therefore, patients with CMVD constitute a considerable population of patients with STEMI undergoing primary PCI and deserve more attention.

Wire-based IMR is related to the presence and severity of microvascular obstruction (MVO) and infarct size, as assessed using cardiovascular magnetic resonance [17, 18]. Similarly, this study found that patients with a caIMR in the IRA of >40 had significantly increased serum peak troponin I and CK-MB levels compared with those with a caIMR in the IRA of ≤40. An elevated caIMR in the IRA implies more severe myocardial damage, as reflected by elevated cardiac enzyme or marker levels [19, 20]. MVO in the IRA results in microinfarcts followed by an inflammatory response, which could contribute to increased myocyte death, thus, leading to increased myocardial enzyme release [21, 22].

In this study, the caIMR in the IRAs was significantly higher than in the reference vessels. Several potential explanations exist for the difference in the caIMR between the IRA and reference vessels. First, coronary microembolization due to the spontaneous or interventional rupture of an epicardial coronary atherosclerotic plaque may cause physical obstruction in the coronary microvessels and induce CMVD in the infarct territory subtended by the IRA [23]. Second, reperfusion could paradoxically impact the microvascular function status, namely, reperfusion injury [24, 25]. Reperfusion injury in STEMI patients is considered a consequence of a series of pathophysiological mechanisms, including MVO, intramyocardial hemorrhage, endothelial damage, and extravascular compression of the microvasculature [26]. Third, the activation of inflammation, release of oxygen-derived free radicals, and disruption of the coagulation pathway could worsen CMVD after reperfusion [14, 27]. Otherwise, we observed a higher proportion of aspiration thrombectomy in the caIMR >40 group, for the greater thrombus burden on visual assessment. This could potentially contribute to the worse microvascular function post PCI, due to the microembolus derived from thrombus debris.

It was noteworthy that in this study the caIMR >40 group had greater caIMR in the reference vessels than the caIMR ≤40 group. As far as we know, this is the first report evaluating the microcirculation function of non-IRAs indicated by caIMR in patients with STEMI undergoing primary PCI. There might be concerns regarding the significant differences in caIMR in the non-culprit vessel territory subtended by the non-IRA between the two groups. We presume that a series of pathophysiological processes in reperfusion therapy trigger global coronary microvascular dysfunction. Furthermore, as indicated by an increased caIMR in the reference vessel, patients in the caIMR >40 group were more likely to have baseline coronary microvascular dysfunction.

In our study, the caIMR >40 group had a markedly increased rate of MACEs compared with the caIMR ≤40 group, which was mainly attributed to a higher rehospitalization rate due to CHF, MI, or angina. In the adjusted analysis for various related variables, the caIMR >40 group had a significantly increased risk of MACEs, regardless of short-term or long-term outcomes. A caIMR in the IRA >40 was identified as an independent predictor of the primary outcome, with an approximately 2–3-fold increase in the risk of MACEs among patients with STEMI after primary PCI. These results align with those of a previous study and thereby support the independent prognostic role of the caIMR in primary PCI [9, 28]. Coronary microvascular dysfunction, indicated by a caIMR in the IRA of >40, was associated with a malignant outcome in patients with STEMI undergoing primary PCI.

### Study Limitations

There are some limitations in this study. Firstly, this study was a single-center observational study with a small sample size, and its findings were not robust due to the absence of a control group. However, we measured the caIMR in the reference vessels to perform a self-control analysis. The limited number of events could lead to overfitting in the multivariable Cox survival analysis model. Therefore, a prospective, randomized trial with larger populations is necessary. Secondly, more than 50% of patients with multivessel disease were included in this study, and it was difficult to achieve complete revascularization in cases of primary PCI. Although we performed multivariate analysis to control for the potential confounding impact of multivessel disease, incomplete revascularization has been associated with an unfavorable prognosis [29]. Thirdly, although it has been reported that the severity of epicardial stenosis does not influence coronary microcirculatory resistance, the caIMR measurement was pressure-dependent, which was closely related to ${\mathrm{Pd}}_{\text{hyp}}$ and $V_{\text{diastole}}$. As previously described in the Methods section, severe stenosis in the target vessels may result in low blood pressure during the peri-procedure, potentially affecting the velocity and pressure in the distal vessel. However, we used a corrected IMR following the Yong formula in cases with severe coronary stenosis to reveal the actual coronary microvascular function. Fourthly, in the present study, we used a caIMR of >40 after primary PCI to reflect the severe microcirculatory impairment. A cutoff value of >40 was referenced from a relevant study; this was a pressure‒temperature wire-derived value [4, 5]. Whether a wire-derived IMR cutoff value of >40 can be translated into caIMR for primary PCI deserves further study. Fifthly, there were some uninterpretable studies, meaning further studies are warranted to explore the potential explanation.

## 5. Conclusions

CMVD in patients with STEMI undergoing primary PCI is not a rare situation. A caIMR in the IRA of >40 implied more myocardial damage, and the caIMR was significantly higher in the IRAs than in the non-IRAs. The caIMR in the reference vessels of the caIMR >40 group was greater than in the caIMR ≤40 group. A caIMR in the IRA of >40 was associated with a higher risk of poor outcomes in patients with STEMI undergoing primary PCI. The clinical implications of a caIMR in patients with STEMI warrant further studies to clarify its diagnostic performance and prognostic stratification in primary PCI.

## Data Availability

The datasets analyzed in the current study are available from the corresponding author upon reasonable request.

## Abbreviations

ACEI, angiotensin-converting enzyme inhibitor; ARB, angiotensin II receptor antagonist; BMI, body mass index; CK-MB, creatine kinase-myocardial band; CI, confidence interval; CHF, congestive heart failure; caIMR, coronary angiography-derived index of microcirculatory resistance; CMVD, coronary microvascular dysfunction; DBP, diastolic blood pressure; eGFR, estimated glomerular filtration rate; HR, hazard ratio; IMR, index of microcirculatory resistance; IRA, infarct-related artery; LAD, left anterior descending artery; LCX, left circumflex; LDL-C, low-density lipoprotein cholesterol; LM, left main; LVEF, left ventricular ejection fraction; MACEs, major adverse cardiovascular events; MVO, microvascular obstruction; MVD, multivessel disease; MI, myocardial infarction; PCI, percutaneous coronary intervention; RCA, right coronary artery; SBP, systolic blood pressure; STEMI, ST-segment elevation myocardial infarction; TIMI, thrombolysis in MI; TVR, target vessel revascularization; WBC, white blood cell.
